# Molecular Epidemiology Reveals Low Genetic Diversity among *Cryptococcus neoformans* Isolates from People Living with HIV in Lima, Peru, during the Pre-HAART Era

**DOI:** 10.3390/pathogens9080665

**Published:** 2020-08-18

**Authors:** Nathalie van de Wiele, Edgar Neyra, Carolina Firacative, Felix Gilgado, Carolina Serena, Beatriz Bustamante, Wieland Meyer

**Affiliations:** 1 Molecular Mycology Research Laboratory, Centre for Infectious Diseases and Microbiology, Westmead Clinical School, Sydney Medical School, Faculty of Medicine and Health, Marie Bashir Institute for Infectious Diseases and Biosecurity, The University of Sydney, Research and Education Network-Westmead Hospital, Westmead Institute for Medical Research, Westmead, NSW 2145, Australia; n.vdwiele@bio-aware.com (N.v.d.W.); cfiracative@gmail.com (C.F.); felix.gilgado@gmail.com (F.G.); carolserena@gmail.com (C.S.); 2 Unidad de Genόmica, Laboratorios de Investigaciόn y Desarrollo, Universidad Peruana Cayetano Heredia, Lima 15102, Peru; edgar.neyra@upch.pe; 3 Facultad de Medicina, Universidad Peruana Cayetano Heredia, Lima 15102, Peru; 4 Studies in Translational Microbiology and Emerging Diseases (MICROS) Research Group, School of Medicine and Health Sciences, Universidad del Rosario, Bogota 111221, Colombia; 5 Institut d’Investigació Sanitària Pere Virgili. Hospital Universitari Joan XXIII, 43005 Tarragona, Spain; 6 Instituto de Medicina Tropical Alexander von Humboldt, Universidad Peruana Cayetano Heredia, Lima 15102, Peru; ana.bustamante@upch.pe

**Keywords:** *Cryptococcus neoformans*, HIV positive patients, Peru, genotyping, MLST

## Abstract

Cryptococcosis, a mycosis presenting mostly as meningoencephalitis, affecting predominantly human immunodeficiency virus (HIV)-infected people, is mainly caused by *Cryptococcus neoformans*. The genetic variation of 48 *C. neoformans* isolates, recovered from 20 HIV-positive people in Lima, Peru, during the pre-highly active antiretroviral therapy (HAART) era, was studied retrospectively. The mating type of the isolates was determined by PCR, and the serotype by agglutination and *CAP59*-restriction fragment length polymorphism (RFLP). Genetic diversity was assessed by *URA5*-RFLP, PCR-fingerprinting, amplified fragment length polymorphism (AFLP), and multilocus sequence typing (MLST). All isolates were mating type alpha, with 39 molecular type VNI, seven VNII, corresponding to *C. neoformans* var. *grubii* serotype A, and two VNIII AD hybrids. Overall, the cryptococcal population from HIV-positive people in Lima shows a low degree of genetic diversity. In most patients with persistent cryptococcal infection, the same genotype was recovered during the follow-up. In four patients with relapse and one with therapy failure, different genotypes were found in isolates from the re-infection and from the isolate recovered at the end of the treatment. In one patient, two genotypes were found in the first cryptococcosis episode. This study contributes data from Peru to the ongoing worldwide population genetic analysis of *Cryptococcus*.

## 1. Introduction

Cryptococcosis is an opportunistic mycosis, which has a worldwide distribution and is caused by the encapsulated yeasts of the *Cryptococcus neoformans* and *C. gattii* species complexes [1]. *C. neoformans*, which is found worldwide, has long been classified into two varieties, *C. neoformans* var. *grubii* (serotype A) and *C. neoformans* var. *neoformans* (serotype D), and AD hybrids (serotype AD). The second species, *C. gattii* (serotypes B and C), is found mainly in tropical and subtropical areas, although its prevalence has increased in the last two decades in temperate climates such as British Columbia, Canada, the Pacific Northwest of the United States and some regions in Europe [2,3]. However, the majority of human cryptococcosis cases are caused by *C. neoformans* var. *grubii* (serotype A), comprising 95% of all clinical isolates [1].

Within the two species complexes, at least eight major molecular types have been defined based on PCR-fingerprinting, amplification fragment length polymorphism (AFLP) analysis, and restriction fragment length polymorphism (RFLP) analysis of the *URA5* gene. These molecular types are VNI, VNII and VNB for *C. neoformans* var. *grubii*, VNIII for the AD hybrid, VNIV for *C. neoformans* var. *neoformans* and VGI to VGIV for *C. gattii* [4,5,6]. However, depending on the typing method used, the results may not be completely comparable between laboratories. Therefore, the Working Group for Genotyping of *C. neoformans* and *C. gattii* of the International Society for Human and Animal Mycology (ISHAM) developed a consensus multilocus sequence typing (MLST) scheme containing seven unrelated genetic loci [7].

Cryptococcal disease is acquired by inhalation and deposition of the infectious propagules/yeasts in the lungs and subsequent invasion of the central nervous system (CNS) and other organs. Similar to other infections of the lungs, cryptococcosis can occur as primary infection with the formation of pulmonary granulomas that can restrain infection or eradicate the yeast [8]. When dormant yeasts cells remain in the lungs, reactivation and clinical disease can occur in the setting of immune deficiency [9]. In addition, there are multiple potential causes of relapse of cryptococcal disease. Amongst them, the choice and activity of the initial antifungal treatment, the non-adherence to the secondary prophylaxis, and the lack of antiretroviral treatment (ART), contribute to a higher risk of relapse [10]. However, although the isolates of the primary infection and the ones obtained during a relapse are in most cases reported to be the same, there are some studies that indicate the possibility of multiple simultaneous infections with different strains, serotypes or even species [11,12,13,14].

Regarding the epidemiology of cryptococcosis worldwide, in over 80% of cases, HIV is the underlying medical condition and detection of the yeast in the body is therefore in many patients the first indication of AIDS [15]. However, many immunocompetent people are probably exposed but are asymptomatic or have only mild symptoms [16]. Among the isolates from AIDS patients, the molecular type VNI is the most common [1,5].

Since the emergence of the HIV epidemic, the number of patients with cryptococcal infections increased dramatically [17]. By the end of 2018, almost 38 million people were living with HIV worldwide, with approximately 79,000 living in Peru, mostly in Lima and Callao, the capital area [18,19,20]. In 2018, 5911 new HIV cases, including 1362 in the AIDS stage, were diagnosed in Peru, with an estimated incidence of cryptococcal infection of 500 cases per year [15,18]. In addition, the response of cryptococcosis in AIDS patients to antifungal treatment is in general relatively poor [13,21]. After introducing highly active antiretroviral treatment (HAART), infections of cryptococcosis decreased significantly in the developed countries, however, in countries where access to HAART and health care services is limited, the number of cryptococcal infections is still extremely high [22]. Although in Peru the HAART program was implemented in May 2004, cryptococcal disease is still developed in those HIV infected individuals who delay seeking medical assistance, as such it is still an important opportunistic infection [23].

The purpose of the current study was to genetically characterize the cryptococcal population recovered from people living with HIV in Lima, Peru, collected during the pre-HAART era, to establish whether a second infection of a patient is due to a relapse or a new infection, and to place the Peruvian genotypes into the global molecular epidemiology map of cryptococcosis.

## 2. Results

### 2.1. Mating Type and Serotype Analysis

All isolates from Peru were mating type alpha (Table 1). According with the slide agglutination test, all studied isolates were identified as serotype A. However, when the serotype was determined by RFLP analysis of the *CAP59* gene, two of the studied isolates (WM 05.515 and WM 05.516), which were obtained from the same individual, were serotype AD (Table 1).

### 2.2. Major Molecular Type Determination

**Figure 1:**
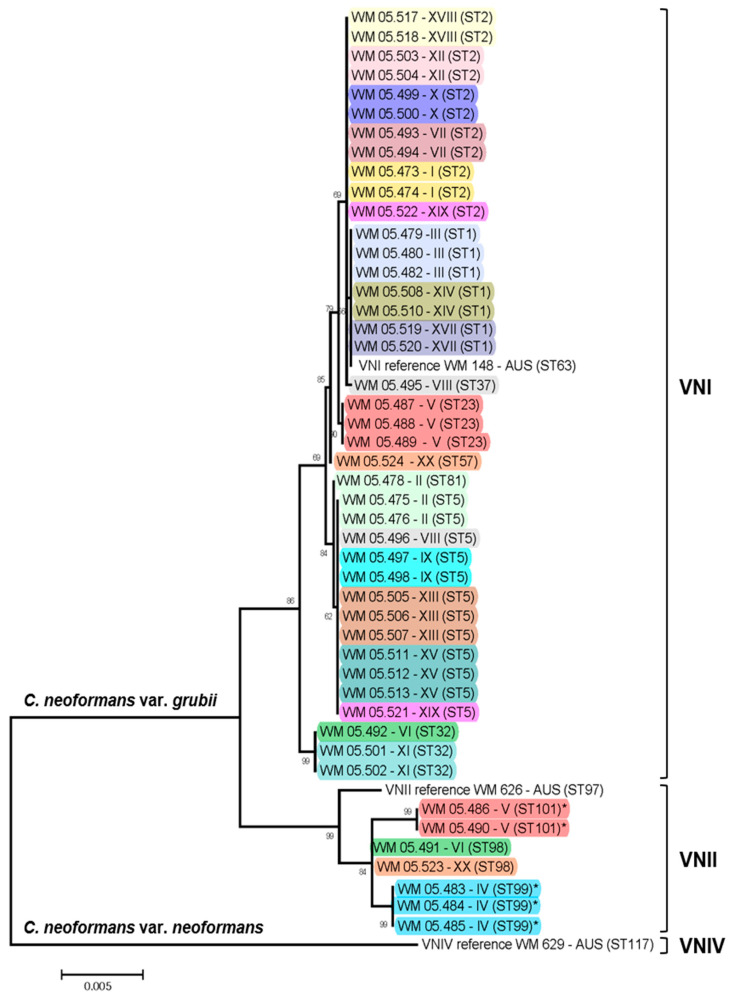
Genetic relationship of clinical *Cryptococcus neoformans* VNI and VNII isolates from Peru. Sequence types (ST) identified for the first time in this study are indicated with an asterisk (*). Bootstrap values are shown on top of the branches. Isolate numbers are followed by the patient number (see Table 1) and the ST. Reference strains from Australia (AUS) for the molecular types VNI, VNII and VNIV are shown.

RFLP analysis of the *URA5* gene and M13 PCR-fingerprinting analysis identified 39 of the studied isolates as major molecular type VNI, seven as VNII and two as the hybrid VNIII (Table 1). From 17 patients, the same molecular type was recovered in all serial isolates. For two patients (VI, and XX,) the baseline isolate was VNII and the isolate recovered from a relapse was VNI (Table 1, Figure 1). From one patient (V) five samples were taken; the first baseline isolate from sputum was VNII, the following two isolates, from CSF and sputum, before the patient had any treatment, and the isolate recovered after seven days of treatment, were VNI. However, the last isolate obtained at the day 14 of the treatment was VNII (Table 1, Figure 1). AFLP analysis identified three groups of isolates, AFLPI, AFLPII, and AFLPIII, corresponding to the molecular types VNI, VNII, and VNIII, respectively (Table 1).

### 2.3. MLST Typing

**Figure 2:**
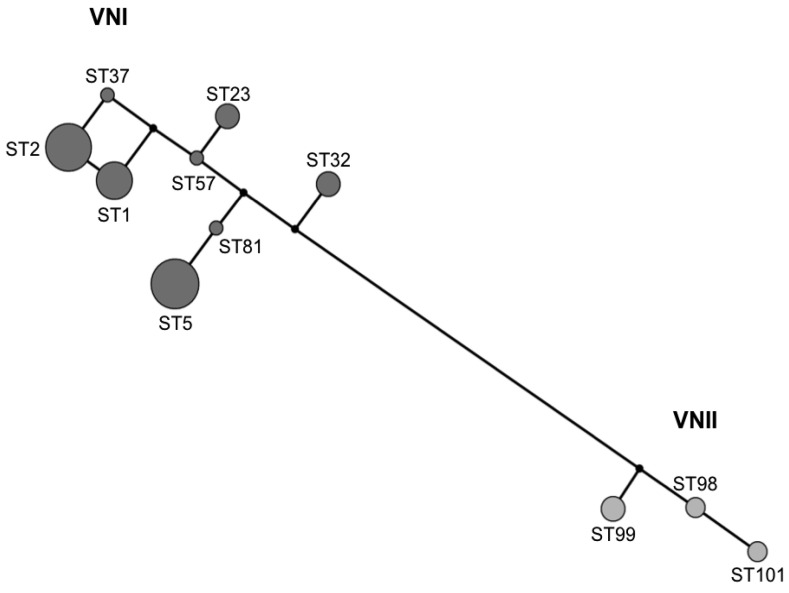
Gene network analysis of clinical *Cryptococcus neoformans* VNI and VNII isolates from Peru based on the data obtained from the 7 genetic loci of the ISHAM MLST consensus scheme for *C. neoformans* and *C. gattii*. Sequence type (ST) numbers are indicated. Circle size is proportional to the frequency of each ST.

Eight STs were identified among the 39 VNI isolates and three STs among the seven VNII isolates. Among the VNI isolates, ST5 and ST2 were the most prevalent with 12 (30.77%) and 11 (28.20%) isolates, respectively. ST99 and ST101, both VNII, were identified for the first time in this study (Table 2, Figure 1). Overall, the population of *C. neoformans* from Peru showed a low genetic diversity (D = 0.17), with the molecular type VNII being slightly more diverse than the molecular type VNI (D = 0.35 vs. D = 0.22, respectively). Network analysis revealed a clear separation and a low recombination between the isolates (Figure 2).

### 2.4. Recombination and Clonality

The analysis of the VNI (IA = 1.8913, rBarD = 0.38408) and VNII (IA = 2.13934, rBarD = 0.538629) population rejected the null hypothesis of recombination (*p* < 0.001), which is also observed in the network analysis (Figure 2). Taken together, these analyses are consistent with the low genetic diversity found in the global population of *C. neoformans*.

## 3. Discussion

This is the first retrospective study assessing the genetic diversity of sequential isolates of *C. neoformans* obtained from HIV positive patients from Lima, Peru during the pre–HAART era. As observed globally among HIV positive patients, in Lima, the prevalence of male patients (70%) among the studied population was higher, even before the beginning of the AIDS epidemic [43]. Similarly, the predominance of *C. neoformans* var. *grubii*, mating type alpha, molecular type VNI (81.25%) among the studied isolates from AIDS patients agrees with the worldwide distribution of cryptococcal species, mating types and molecular types causing cryptococcosis [1,5,44]. This study identified however the less common molecular type VNII (14.58%), which has been reported in the neighboring countries Brazil, Colombia and Chile, and, for the first time in Peru, the molecular type VNIII (4.17%), which has been identified very rarely in the region [44]. The molecular type VNIV, which is commonly identified in Europe [11,28,31], was not identified.

The utilization of RFLP analysis of the *CAP59* gene for the determination of the serotype of the isolates was of great value in this study, as the methodology using agglutination with the Crypto-Check Kit did not identify correctly the serotype AD of the hybrid strains. Contradictory serotyping results for AD hybrid strains have been reported previously [45], which could be due to either unspecific results obtained by the serological method used or alterations in the studied strain itself, resulting in phenotypic changes [46].

Although the resolution to distinguish among isolates of the same species differed among the methods used in this study, it was possible to obtain comparable results, concerning major molecular type identification, by using RFLP analysis of the *URA5* gene, PCR-fingerprinting, AFLP and MLST. However, MLST showed once again to have the greatest resolution power when identifying strain differences. Genotyping the isolates using MLST showed that most of the persistent cryptococcal disease cases were due to a relapse caused by the baseline isolate that was initially recovered when cryptococcosis was first diagnosed and before starting treatment. Similar findings were previously reported from 33 and 30 HIV positive patients with cryptococcosis from the USA, and Brazil, respectively [13,47]. However, in five patients from Peru, the serial isolates (‘baseline’, ‘failure’, ‘relapse’ or ‘in treatment’) differed in the genotype (patients II, VIII and XIX) and in the molecular type (patients VI and XX) identified, which suggests that in many cases, patients can either be infected by multiple strains or be re-infected with a new isolate (Table 1). A study in Cuba already suggested the possibility of multiple simultaneous infections with different strains, serotypes or even species [48]. In the current study, one patient (V) showed two different molecular types among the baseline and consecutive (7th and 14th day) isolates recovered from two different samples, sputum and CSF, which could be explained by simultaneous mixed infections. In France, mixed infections with different mating types, serotypes, ploidies and genotypes have been reported during the same episode of cryptococcosis in 20% of the patients, which suggested that multiple strains could be exogenously acquired from the environment, either simultaneous or sequentially [11]. Because most of the times only one single colony is selected from the clinical samples in the microbiology diagnostics laboratory prior to the molecular analysis, as such the number of mixed infections is most likely globally underestimated.

The genotypic characterization of *C. neoformans* isolates from Peru also showed that this population is relatively homogeneous, since half of the studied isolates were represented by only two sequence types (Figure 1) and it presents a low genetic diversity (D = 0.17). The low degree of recombination between isolates (Figure 2), which could be explained by the presence of a unique mating type alpha among the isolates that does not contribute to sexual reproduction, also supports the low degree of genetic variation among the *C. neoformans* population, as reported elsewhere [33,39]. This is in strong contrast to the identification of both mating types a and alpha among cryptococcal isolates, recovered from AIDS patients in Botswana, Africa, which revealed a unique and highly diverse genetic sub-population (VNB) and proved evidence for sexual recombination [24,49].

In the global context, the genotypes identified in Peru do not represent a unique population but rather genotypes that are found in other regions of the world (Table 2). The most common genotype identified in Peru (ST5) is the genotype with the greatest numbers of isolates worldwide. It has been recovered in large proportions in Asia and sporadically in Europe, Africa, South America and the USA (Table 2). Interestingly, this genotype has been found not only in clinical isolates, but also in environmental and veterinarian isolates [36].

With almost the same proportion as ST5, ST2 was the second most common ST in Peru. This ST, identified previously in Argentina, Brazil Colombia, France, Germany, Malawi, South Africa, Tanzania, and the USA (Table 2), has been mostly identified in clinical isolates. Only few isolates with ST2 from pigeon droppings have been recovered in the USA [26], which reaffirms the saprophytic source of *C. neoformans*.

In conclusion the current study investigating *C. neoformans* var. *grubii* VNI, VNII and VNIII isolates from HIV positive patients from Lima, Peru, revealed that this population showed mainly an origin from a clonal population structure with a low level of genetic recombination. It also revealed that persistent infection or relapse can be caused by the original isolate, an isolates which may had been originally overlooked, as in most cases only a single colony is usually picked from the primary isolation plate for the diagnostic workup in a clinical laboratory, or by re-infection with a new strain. This study generated valuable data from Peru to further expand the picture of the global *C. neoformans* and *C. gattii* population genetic analysis.

## 4. Materials and Methods 

### 4.1. Isolates

A total of 48 *C. neoformans* isolates from 14 (70%) male and 6 (30%) female, HIV positive patients from Peru were collected at the Instituto de Medicina Tropical Alexander von Humboldt-Universidad Peruana Cayetano Heredia in Lima, Peru, between June 1997 and June 2002. From the isolates, 46 (95.83%) were recovered from cerebrospinal fluid (CSF) and two (4.17%) from sputum. The age of the patients at baseline ranged from 22 to 74 years (Table 1). All patients received amphotericin B deoxycholate 0.7 mg/kg/day as induction treatment for 2–3 weeks, followed by fluconazole 400 mg daily for 8 weeks as consolidation treatment. After that, they continued with fluconazole 200 mg per day as maintenance treatment.

From each patient at least two consecutive isolates were taken (Table 1). The primary ‘baseline’ isolate was recovered at the first episode, before initiating the treatment; the ‘failure’ isolate was recovered when sterility of CSF was not achieved at day 70th of antifungal treatment; a ‘relapse’ isolate was recovered after the patient had originally been confirmed as cured with a culture negative for *Cryptococcus* spp., and the ‘in treatment’ isolate was recovered during any day of antifungal treatment for cryptococcosis. All the isolates were collected during the pre-HAART era.

Eight strains from the Molecular Mycology Research Laboratory at Sydney Medical School—Westmead Institute for Medical Research, University of Sydney, Australia, were used as references of the major molecular types. These reference strains were WM 148 (*C. neoformans* var. *grubii*, VNI, serotype A), WM 626 (*C. neoformans* var. *grubii*, VNII, serotype A), WM 628 (AD hybrid, VNIII, serotype AD), WM 629 (*C. neoformans* var. *neoformans*, VNIV, serotype D), WM 179 (*C. gattii*, VGI, serotype B), WM 178 (*C. gattii*, VGII, serotype B), WM 175 (*C. gattii*, VGIII, serotype B) and WM 779 (*C. gattii*, VGIV, serotype C) [5].

### 4.2. DNA Extraction

For genomic DNA extraction, the isolates were cultured on Sabouraud dextrose agar at 37 °C for 48 h. High molecular weight DNA was then extracted as described previously [50], with some modifications. Half an inoculation loop of cells from the culture was transferred into a microcentrifuge tube and stored at −20 °C overnight. The cells were then incubated at 65 °C for 1 h with 500 µL of lysis buffer (0.5 g SDS, 1.4 g NaCl, 0.73 g EDTA, 20 mL Tris-HCL 1M) and 5 µL of 2-mercaptoethanol. After incubation, 500 µL of phenol:chloroform:isoamyl alcohol (v:v:v 25:24:1) were added and mixed thoroughly for 2 min to obtain a homogeneous suspension, and centrifuged at 14,000 rpm for 15 min. The upper aqueous layer was removed and mixed with an equal volume of isopropanol and the DNA was precipitated at −20 °C overnight. Thereafter, the mixture was centrifuged at 14,000 rpm at 4 °C for 15 min, and the supernatant was discarded. The pellet was washed with 500 µL of 70% aqueous solution of ethanol and centrifuged at 14,000 rpm for 15 min followed by air-drying. The DNA pellet was resuspended in 50–100 µL sterile distilled water. DNA concentration was measured using the BioPhotometer (Eppendorf) by reading the UV absorbance at 260 nm and adjusted to a final concentration of 10 ng/µL.

### 4.3. Mating Type Determination

The mating type of the isolates was determined by PCR as previously described [51], with some modifications. The total volume of the PCR mix was 25 µL containing 1× PCR buffer, 0.2 mM of dNTPs, 1.25 U of BIO TAQ™ DNA polymerase (BIOLINE), 3 mM MgCl_2_, 24 ρmol of primer MfalphaU (5′ TTC ACT GCC ATC TTC ACC ACC 3′) and 31.6 ρmol of primer MfalphaL (5′ TCT AGG CGA TGA CAC AAA GGG 3′) for MATalpha, or 1.5 mM MgCl_2_, and 100 ρmol for each primer Mfa2U (5′ ACA CCG CCT GTT ACA ATG GAC 3′) and Mfa2L (5′ CAG CGT TTG AAG ATG GAC TTT 3′), for MATa. The PCR products were electrophoresed in a 2.5% agarose gel (2.5% in Tris-borate-EDTA buffer) with 0.5 µL/mL ethidium bromide, at 100 V for 30 min. The strains WM 148 and WM 06.38 were used as reference for mating type alpha and a, respectively. The gels were visualized by UV light and the mating types were assigned visually by comparing the studied isolates with the reference strains.

### 4.4. Serotyping

The serotype of the isolates was firstly determined with the slide agglutination test using the Crypto-Check Kit according to the manufacturer’s instructions (Iatron Labs. Tokyo, Japan). In addition, serotyping via RFLP of the *CAP59* gene was carried out. The *CAP59* gene was amplified in a total volume of 50 µL, as previously published [52], using the primers CH-Cap59-F (5′ CCT TGC CGA AGT TCG AAA CG 3′), and CH-Cap59-R (5′ AAT CGG TGG TTG GAT TCA GTG T 3′) (Eurogentec, Liege, Belgium). Each reaction included 1× PCR, 1 µM of each primer, 0.2 mM of dNTPs, 1.5 U of BIO TAQ™ DNA polymerase (BIOLINE), and 3 mM MgCl_2_. The PCR products were double digested with the restriction enzymes *BsmFI* and *HpaII* as instructed by the manufacturer (New England BioLabs Inc. Ipswich, MA, USA). A 3% agarose electrophoresis gel (Tris-borate-EDTA buffer) with 0.5 µL/mL ethidium bromide was used to separate the fragments and run at 80 V. Serotype was determined according with the number and size of the CAP59 gene fragments after restriction.

### 4.5. Molecular Typing 

The major molecular type of the isolates was identified by RFLP of the *URA5* gene, and the genetic diversity was accessed using M13 PCR-fingerprinting, AFLP and MLST. 

*RFLP analysis of the URA5 gene* was done as follows: The *URA5* gene was amplified as described previously [5], with some changes. Each reaction contained 30 ng of genomic DNA, 1 x PCR buffer (10 mM Tris-HCl, pH 8.3, 50 mM KCl, 1.5 mM MgCl_2_; Applied Biosystems, Foster City, CA, USA), 0.2 mM of dNTPs (Roche Diagnostics GmbH), 3 mM magnesium acetate, 50 ng of each primer URA5 (5′ ATG TCC TCC CAA GCC CTC GAC TCC G 3′) and SJ01 (5′ TTA AGA CCT CTG AAC ACC GTA CTC 3′) and 2.5 U AmpliTaq DNA polymerase (Applied Biosystems), in a final volume of 50 µL. The amplification product was double digested with 6.7 U *HhaI* and 3 U *Sau96I* (New England Biolabs). The reaction was incubated at 37 °C for at least 3 h in a thermal heating block, and the RFLP fragments were separated on a 3% agarose gel containing 0.2 µg/mL ethidium bromide at 2.45 V/cm for 4 h. The fragments were visualized under UV light and the major molecular types were identified by comparison with the eight standard strains representing the molecular types VNI-VNIV and VGI-VGIV.

*PCR-fingerprinting analysis* was conducted with the minisatellite-specific primer M13 (5′ GAG GGT GGC GGT TCT 3′), was used as single primer in the PCR. PCR-fingerprinting was performed as previously described [6]. Amplification products were separated on a 1.4% agarose gel at 80 V for 14 cm. To normalize the gels, a 1 kb molecular size marker (GIBCO-BRL, Life technologies, Gaithersburg, Rockville, MD, USA) was used in each gel in several lanes and visualized under UV light. Molecular types were assigned according to the major bands when compared with the patterns of the eight molecular type reference strains. All visible bands were included in the analysis despite their intensity.

*AFLP* was performed according to the AFLP Analysis System for Microorganisms protocol (AFLP microorganism primer kit; Invitrogen Life Technologies, Carlsbad, Calif.). The first PCR was performed with the two pre-selective primers EcoRI core sequence and MseI core sequence, under the following conditions: 20 cycles of 30 s at 94 °C, 60 s at 56 °C, and 60 s at 72 °C. A second PCR was performed using selective fluorescent primers EcoRI-AAC and primer MseI-CAG. Conditions were as follows: a denaturation step for 30 s at 94 °C, an annealing step for 1 min, and an extension step for 1 min at 72 °C. The annealing temperature in the first cycle was 65 °C; for each of the next 12 cycles, the annealing temperature was reduced further by 0.7 °C; for the remaining 23 cycles, it was kept at 56 °C. Other combinations such as EAAC and MCAA were used in order to resolve doubts in the AFLP patterns generated. For this study, the obtained AFLP patterns were grouped according to their major bands with the software package BioloMICS version 8.8.1.11 (Bio-Aware, Hannut, Belgium) and AFLP type numbers, AFLPI, AFLPII and AFLPIII, were assigned to each major group corresponding to the major molecular types VNI, VNII and VNIII, respectively.

*MLST* was carried out on the haploid isolates (VNI and VNII), using the ISHAM consensus MLST scheme for *C. neoformans* and *C. gattii*, including seven independent genetic loci [7]. These loci are dispersed over six different chromosomes and include six housekeeping genes (*CAP59*, *GPD1*, *LAC1*, *PLB1*, *SOD1*, and *URA5*) and the intergenic spacer region IGS1 of the rDNA gene cluster. Each amplification mixture was set up in a 50 µL reaction volume containing: 1× PCR buffer, 100 ng DNA, 2 mM MgCl_2_, 7.5 pmol of each primer, 0.2 mM of dNTPs and 2.5 U BIO TAQ™ DNA polymerase (BIOLINE). Amplifications conditions were followed as described previously [7]. Sequences were obtained commercially by Macrogen Inc., Korea, edited with the software package BioloMICS version 8.8.1.11 (Bio-Aware, Hannut, Belgium) and aligned with the program Mega version 7.0 [53]. For each unique sequence of each MLST locus, an allele type (AT) was assigned, followed by the assignment of a sequence type (ST) for each unique allele type combination. Allele types and sequence types were assigned in accordance with the MLST database of the Molecular Mycology Research Laboratory, available at http://mlst.mycologylab.org. All allele types per loci were deposited in the MLST database and in GenBank under the accession numbers KF792024 to KF792048 (Table S1). With the concatenated sequences, a dendrogram showing the genetic relationships between the isolates was constructed with the program Mega version 7.0 [53], based on maximum likelihood analysis. The three reference strains of the haploid major molecular types VNI, VNII and VNIV, from Australia were added to the sequence analysis (see above). The strain WM 629 (VNIV) was assigned as an out-group. The genetic diversity of the isolates was assessed by calculating the Simpson’s Diversity index (D) for the whole *C. neoformans* population and for each molecular type VNI and VNII [54]. Network analysis of the STs identified in this study was performed with the program Network 4.6 (http://www.fluxus-engineering.com) using the median joining algorithm.

### 4.6. Recombination and Clonality

The importance of clonal versus sexual reproduction was assessed for each haploid molecular type by calculating the index of association (IA) and rBarD, which is a modification of IA that removes the dependency on the number of loci. IA and rBarD were calculated using the program Multilocus version 1.3b [55], either for the full data set or once the data set was reduced to unique haplotypes or STs (clone-corrected data).

## Figures and Tables

**Table 1:** *Cryptococcus neoformans* isolates recovered from HIV positives patients from Peru, indicating epidemiological and genotypic data.

Patient Number	WM Number	Isolation Point	Date of Isolation	Source ^a^	Age/Gender	*URA5*-RFLP and PCR fingerprinting	AFLP Type	Mating Type/Serotype	*CAP59*	*GPD1*	IGS1	*LAC1*	*PLB1*	*SOD1*	*URA5*	Sequence Type (ST)
I	WM 05.473	Relapse	23/06/97	CSF	26/M	VNI	I	alpha/A	7	1	1	1	1	1	2	2
	WM 05.474	Relapse	27/10/98	CSF		VNI	I	alpha/A	7	1	1	1	1	1	2	2
II	WM 05.475	Baseline	20/10/99	CSF	74/M	VNI	I	alpha/A	1	3	1	5	2	1	1	5
	WM 05.478	Failure	19/07/00	CSF		VNI	I	alpha/A	1	1	1	5	2	1	1	81
	WM 05.476	Failure	30/10/00	CSF		VNI	I	alpha/A	1	3	1	5	2	1	1	5
III	WM 05.479	Baseline	29/11/99	CSF	26/M	VNI	I	alpha/A	7	1	1	1	1	1	1	1
	WM 05.480	Failure	16/06/00	CSF		VNI	I	alpha/A	7	1	1	1	1	1	1	1
	WM 05.482	Failure	17/08/00	CSF		VNI	I	alpha/A	7	1	1	1	1	1	1	1
IV	WM 05.483	Baseline	18/01/00	CSF		VNII	II	alpha/A	2	9	38	11	11	16	15	99 *
	WM 05.484	Relapse	28/04/00	CSF	23/F	VNII	II	alpha/A	2	9	38	11	11	16	15	99 *
	WM 05.485	Failure	3/06/00	CSF		VNII	II	alpha/A	2	9	38	11	11	16	15	99 *
V	WM 05.486	Baseline	13/03/00	Sputum ^b^		VNII	II	alpha/A	2	10	39	8	12	16	33	101 *
	WM 05.487	Baseline	11/09/00	CSF		VNI	I	alpha/A	7	1	1	2	1	1	2	23
	WM 05.488	Baseline	13/09/00	Sputum ^b^	28/M	VNI	I	alpha/A	7	1	1	2	1	1	2	23
	WM 05.489	7th day treatment	18/09/00	CSF		VNI	I	alpha/A	7	1	1	2	1	1	2	23
	WM 05.490	14th day treatment	25/09/00	CSF		VNII	II	alpha/A	2	10	39	8	12	16	33	101 *
VI	WM 05.491	Baseline	2/01/01	CSF	38/M	VNII	II	alpha/A	2	9	14	8	11	16	4	98
	WM 05.492	Relapse	15/06/01	CSF		VNI	I	alpha/A	1	1	10	3	4	1	1	32
VII	WM 05.493	Baseline	20/06/01	CSF	22/F	VNI	I	alpha/A	7	1	1	1	1	1	2	2
	WM 05.494	Failure	21/09/01	CSF		VNI	I	alpha/A	7	1	1	1	1	1	2	2
VIII	WM 05.495	Baseline	28/10/00	CSF	52/F	VNI	I	alpha/A	1	1	1	1	1	1	2	37
	WM 05.496	Relapse	6/09/01	CSF		VNI	I	alpha/A	1	3	1	5	2	1	1	5
IX	WM 05.497	Baseline	23/08/01	CSF	40/F	VNI	I	alpha/A	1	3	1	5	2	1	1	5
	WM 05.498	Failure	28/01/02	CSF		VNI	I	alpha/A	1	3	1	5	2	1	1	5
X	WM 05.499	Baseline	19/05/98	CSF	35/M	VNI	I	alpha/A	7	1	1	1	1	1	2	2
	WM 05.500	Failure	5/08/98	CSF		VNI	I	alpha/A	7	1	1	1	1	1	2	2
XI	WM 05.501	Baseline	24/08/98	CSF	27/M	VNI	I	alpha/A	1	1	10	3	4	1	1	32
	WM 05.502	Relapse	26/10/99	CSF		VNI	I	alpha/A	1	1	10	3	4	1	1	32
XII	WM 05.503	Baseline	16/10/98	CSF	23/M	VNI	I	alpha/A	7	1	1	1	1	1	2	2
	WM 05.504	Failure	26/12/98	CSF		VNI	I	alpha/A	7	1	1	1	1	1	2	2
XIII	WM 05.505	Baseline	12/11/98	CSF	22/M	VNI	I	alpha/A	1	3	1	5	2	1	1	5
	WM 05.506	Failure	22/01/99	CSF		VNI	I	alpha/A	1	3	1	5	2	1	1	5
	WM 05.507	Failure	23/03/99	CSF		VNI	I	alpha/A	1	3	1	5	2	1	1	5
XIV	WM 05.508	Baseline	15/01/99	CSF	32/M	VNI	I	alpha/A	7	1	1	1	1	1	1	1
	WM 05.510	Failure	26/03/99	CSF		VNI	I	alpha/A	7	1	1	1	1	1	1	1
XV	WM 05.511	Baseline	3/03/99	CSF	37/M	VNI	I	alpha/A	1	3	1	5	2	1	1	5
	WM 05.513	14th day treatment	18/03/99	CSF		VNI	I	alpha/A	1	3	1	5	2	1	1	5
	WM 05.512	Failure	13/05/99	CSF		VNI	I	alpha/A	1	3	1	5	2	1	1	5
XVI	WM 05.515	Baseline	29/04/99	CSF	28/M	VNIII	III	alpha/AD ^c^								
	WM 05.516	Failure	8/07/99	CSF		VNIII	III	alpha/AD ^c^								
XVII	WM 05.519	Baseline	20/05/00	CSF	26/M	VNI	I	alpha/A	7	1	1	1	1	1	1	1
	WM 05.520	Relapse	9/08/00	CSF		VNI	I	alpha/A	7	1	1	1	1	1	1	1
XVIII	WM 05.517	Baseline	10/08/00	CSF	47/F	VNI	I	alpha/A	7	1	1	1	1	1	2	2
	WM 05.518	Relapse	11/01/01	CSF		VNI	I	alpha/A	7	1	1	1	1	1	2	2
XIX	WM 05.521	Baseline	30/12/00	CSF	29/F	VNI	I	alpha/A	1	3	1	5	2	1	1	5
	WM 05.522	Relapse	1/06/01	CSF		VNI	I	alpha/A	7	1	1	1	1	1	2	2
XX	WM 05.523	Baseline	5/03/01	CSF	41/M	VNII	II	alpha/A	2	9	14	8	11	16	4	98
	WM 05.524	Relapse	1/06/02	CSF		VNI	I	alpha/A	1	1	1	3	1	1	1	57

^a^ CSF: Cerebral spinal fluid. ^b^ Both sputum samples were obtained in two different dates, before treatment. ^c^ Identified as serotype A by agglutination (Iatron Labs. Tokyo, Japan). * Sequence types (ST) identified for the first time in this study. Each allele per locus is represented by a different color.

**Table 2:** Worldwide distribution of the sequence types (ST) of *Cryptococcus neoformans* identified in this study.

Molecular Type	Sequence Type (ST)	Country	Reference
VNI	ST1	France ^a^, Peru, USA,	[24]
VNI	ST2	Argentina, Brazil, Colombia, France, Germany, Malawi, Peru, South Africa, Tanzania, USA	[24,25,26,27,28,29,30]
VNI	ST5	Belgium, Brazil, Colombia, France, Germany, Italy, China, Japan, Korea, Thailand, Kuwait, Peru, Qatar, South Africa, Uganda, Malawi, USA ^b^, Vietnam	[11,24,25,27,28,29,30,31,32,33,34,35,36,37,38,39]
VNI	ST23	Belgium ^a^, Brazil, Colombia, France ^a^, Italy, Germany, Japan ^a^, Kuwait, Korea, Peru, South Africa, Uganda, USA ^a,b^	[11,24,25,28,29,30,36,39,40,41]
VNI	ST32	Brazil, Belgium, China, Colombia, Germany, Japan, Peru, South Africa, USA ^b^, Tanzania, Thailand, Uganda, Vietnam, Zaire	[24,28,29,30,32,36,38]
VNI	ST37	Italy, Peru	[31]
VNI	ST57	China, Peru	[42]
VNI	ST81	Italy, Peru, Thailand	[31,34]
VNII	ST98	Mexico, Peru	[5]
VNII	ST99	Peru	Current Study
VNII	ST101	Peru	Current Study

^a^ Environmental and ^b^ veterinary isolates reported.

## Supplementary Materials

The following are available online at https://www.mdpi.com/2076-0817/9/8/665/s1, Table S1: GenBank accession numbers of all MLST sequences used in this study.
