# Supplementary material for: Molecular Epidemiology Reveals Low Genetic Diversity among Cryptococcus neoformans Isolates from People Living with HIV in Lima, Peru, during the Pre-HAART Era
Source: Pathogens. 2020 Aug 18;9(8):665. doi: 10.3390/pathogens9080665 (PMC7459599; doi:10.3390/pathogens9080665)
Supplement: Supplementary file 1 [file pathogens-09-00665-s001.pdf]

Table S1. GenBank accession numbers of all MLST sequences used in this study.

Loci	Allele	GenBank accession No
CAP59	*CAP59* allele 1	KF792046
	CAP59 allele 2	KF792047
	CAP59 allele 7	KF792048
GPD1	*GPD1* allele 1	KF792042
	GPD1 allele 3	KF792043
	GPD1 allele 9	KF792044
	GPD1 allele 10	KF792045
IGS1	*IGS1* allele 1	KF792049
	IGS1 allele 10	KF792050
	IGS1 allele 14	KF792051
	IGS1 allele 38	KF792052
	IGS1 allele 39	KF792053
LAC1	*LAC1* allele 1	KF792036
	LAC1 allele 2	KF792037
	LAC1 allele 3	KF792038
	LAC1 allele 5	KF792039
	LAC1 allele 8	KF792040
	LAC1 allele 11	KF792041
PLB1	*PLB1* allele 1	KF792031
	PLB1 allele 2	KF792032
	PLB1 allele 4	KF792033
	PLB1 allele 11	KF792034
	PLB1 allele 12	KF792035
SOD1	*SOD1* allele 1	KF792029
	SOD1 allele 16	KF792030
URA5	*URA5* allele 1	KF792024
	URA5 allele 2	KF792025
	URA5 allele 4	KF792026
	URA5 allele 15	KF792027
	URA5 allele 33	KF792028
